# The HOTAIR, PRNCR1 and POLR2E polymorphisms are associated with cancer risk: a meta-analysis

**DOI:** 10.18632/oncotarget.14920

**Published:** 2017-01-31

**Authors:** Haiyan Chu, Yaoyao Chen, Qinbo Yuan, Qiuhan Hua, Xu Zhang, Meilin Wang, Na Tong, Wei Zhang, Jinfei Chen, Zhengdong Zhang

**Affiliations:** ^1^ Department of Genetic Toxicology, The Key Laboratory of Modern Toxicology of Ministry of Education, School of Public Health, Nanjing Medical University, Nanjing, China; ^2^ Department of Environmental Genomics, Jiangsu Key Laboratory of Cancer Biomarkers, Prevention and Treatment, Collaborative Innovation Center for Cancer Personalized Medicine, Nanjing Medical University, Nanjing, China; ^3^ Department of Oncology, The Affiliated Nanjing First Hospital, Nanjing Medical University, Nanjing, China; ^4^ Department of Urology, Huaiyin Hospital of Huai’an City, Huai’an, China; ^5^ Department of Urology, The First Affiliated Hospital of Nanjing Medical University, Nanjing, China

**Keywords:** lncRNA, polymorphism, meta-analysis, cancer, epidemiology

## Abstract

Long non-coding RNAs (LncRNAs) have been widely studied and aberrant expression of lncRNAs are involved in diverse cancers. Genetic variation in lncRNAs can influence the lncRNAs expression and function. At present, there are many studies to investigate the association between lncRNAs polymorphisms and cancer susceptibility. However, it has no systematic study to evaluate the association. We performed a meta-analysis to summarize the results of common lncRNAs (*HOTAIR*, *PRNCR1*, *POLR2E* and *H19*) polymorphisms on cancer risk, by using the random-effect model to obtain the odds ratio (ORs) and 95% confidence interval (95%CI). We also applied the meta-regression and publication bias analysis to seek the source of heterogeneity and evaluate the stability of results, respectively. The summary results indicated that *HOTAIR* rs920778 increased the cancer risk in recessive model (OR = 1.61, 95% CI = 1.08-2.41, *P*_heterogeneity_<0.001). For *PRNCR1* (rs1016343, rs16901946) and *POLR2E* (rs3787016), we also found the significant association with incresed risk of cancer (all *P*<0.05). However, we did not observe any significant association between *H19* rs2107425 and cancer risk. Our meta-analysis results revealed that these four lncRNAs polymorphisms (*HOTAIR* rs920778, *PRNCR1* rs1016343 and rs16901946, *POLR2E* rs3787016) can contribute to cancer risk. Further studies should confirm these findings.

## INTRODUCTION

Long non-coding RNAs (LncRNAs) are a class of >200 nt in length non-coding RNAs, which are involved in diverse cellular processes, such as cell cycle, apoptosis, epigenetics, and gene expression regulation [1, 2]. LncRNAs have several subtypes mainly according to the relationship between lncRNAs’ location and corresponding protein-coding gene's location, including sense lncRNAs, antisense lncRNAs, intergenic lncRNAs, intronic lncRNAs and bidirectional lncRNAs [3, 4]. With the development of high throughput chip and sequencing technology, a variety of lncRNAs have been investigated, e.g. HOX transcript antisense RNA (HOTAIR) [5], prostate cancer non-coding RNA1 (PRNCR1) [6], RNA polymerase II polypeptise E gene (POLR2E) [7, 8].

Emerging studies had shown that abnormal expression of lncRNAs was associated with cancer risk. Schmidt and the colleagues reported that the overexpression non-coding metastasis associated lung adenocarcinoma transcript 1 (MALAT1) can increase the lung cancer cell proliferation and migration activity [9]. Similarly, increased expression of HOTAIR contributes to the breast cancer poor prognosis and metastasis [10]. In our previous study, we also identified the antisense of mediator of DNA damage checkpoint protein 1 (MDC1) acting as the suppressor gene involved in bladder cancer [11]. However, the exact etiology is still not clear.

Single nucleotide polymorphisms (SNPs) can influence the gene expression and function, participating in carcinogenesis [12]. Recently, SNPs in *HOTAIR* have been widely studied in multiple cancers [13–21]. Yan et al. performed a population-based study to investigate the association between *HOTAIR* polymorphisms (rs1899663, rs4759314, rs920778) and breast cancer risk, and result indicated that *HOTAIR* rs920778 was associated with the increased risk of breast cancer [14], similarly in esophageal cancer [20]. We also observed that *HOTAIR* rs7958904 can decrease colorectal cancer risk [21]. Besides, we found that lncRNA *PRNCR1* also have been widely investigated in the development of cancer, and *PRNCR1* firstly identified and named in prostate cancer [6, 22–26]. For *PRNCR1*, rs1016343 had been studied widely. It had been reported that Salinas et al. investigated the association between *PRNCR1* rs1016343 and prostate cancer risk in Caucasians and African Americans; however, they only found rs1016343 associated with increased risk of prostate cancer in Caucasians, and no significant association was observed in African Americans [6]. In gastric cancer, Li et al. did not find the significant risk for rs1016343, and find that *PRNCR1* rs13252298, rs7007694, rs1456315 polymorphisms were associated with gastric cancer risk [22]. Additionally, lncRNA *H19* [27–32] and *POLR2E* [7, 8, 33, 34] polymorphisms were studied widely. The published results of *H19* and *POLR2E* polymorphisms with cancer risk were also conflicted. In summary, we noticed that the results of lncRNAs publications were conflicted rather than conclusive. Herein, we conducted a meta-analysis to summarize all eligible case-control studies to evaluate the overall cancer risk and common lncRNAs (*HOTAIR*, *PRNCR1*, *H19*, and *POLR2E*) polymorphisms.

## RESULTS

### Characteristics of included studies

**Figure 1:**
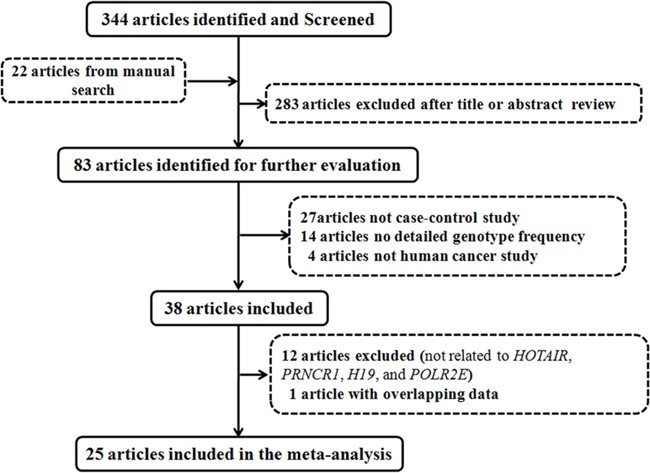
Flow diagram of articles identified with included and excluded criteria

Through searching the PubMed using the key words, we retrieved 344 relevant articles (Figure 1). In addition, we also retrieved 22 articles by manual search of the references of relevant articles. After reviewing the title or abstract, we excluded 283 unrelated articles, leaving 83 articles for further evaluation. We carefully evaluated the full text of articles, and excluded 45 articles (27, not case-control study; 14, no detailed genotype frequency; four, not human cancer study). In these 38 included articles, we observed that a variety of lncRNA genes had been investigated the association with cancer risk, among which lncRNA HOTAIR, PRNCR1, H19, and POLR2E had been widely studied. Thus, in the meta-analysis, we mainly focus on evaluating the association between *HOTAIR*, *PRNCR1*, *H19*, *POLR2E* polymorphisms and cancer risk. Finally, we included 25 relevant articles in this meta-analysis (nine for *HOTAIR*, six for *PRNCR1*, six for H19, and four for *POLR2E*).

As shown in Supplementary Table 1, we summarized the characteristics of the included studies. For *HOTAIR*, there were six studies from China (Asian) and three studies from Turkey (Caucasian), and the source of control of studies mainly was hospital-based population (eight studies). For *PRNCR1*, we also observed that major studies were from Chinese population (67%), and four studies were involved in prostate cancer. For *POLR2E*, there were three studies about prostate cancer, one about esophageal cancer. While for *H19*, studies were including two breast cancer studies, one gastric cancer study, one melanoma study, one ovarian cancer study, and one bladder cancer study. The majority of these studies were matched with age and sex. We found that these included studies mainly used polymerase chain reaction-restriction fragment length polymorphism (PCR-RFLP) assay (8 studies) and TaqMan assay (9 studies) to genotype the polymorphisms. In this meta-analysis, all included SNP genotypes frequency in control was consistent with HWE.

### Quantitative synthesis

We found that the *HOTAIR*, *PRNCR1*, *H19*, *POLR2E* genes were presented very polymorphic, however, not all polymorphic loci had been widely studied. We summarized the common SNPs in Table 1 (the numbers of SNPs >3). For *HOTAIR*, we noticed that there were six studies about rs4759314, five studies about rs920778, and three studies about rs1899663. Herein, we assessed the association between these three SNPs (rs4759314, 920778, and rs1899663) and cancer risk (Table 2). Meta-analysis result showed that rs920778 increased the cancer risk in recessive model (OR = 1.61, 95%CI = 1.08-2.41, *P*_heterogeneity_<0.001) and additive model (1.24, 1.03-1.49, 0.001). For *PRNCR1*, studies mainly reported the relationship of rs1016343 (n = 7), rs13252298 (n = 4), rs7007694 (n = 3), rs16901946 (n = 3), rs1456315 (n = 3) and cancer risk (Table 2). We further evaluated the association between these SNPs and cancer risk, and found that both rs1016343 and rs16901946 increased the risk of cancer in the dominant model (1.27, 1.04-1.53, 0.002; 1.15. 1.02-1.29, 0.573, respectively). In the additive model, we also observed that rs1016343 was associated with a 24% increased risk of cancer (1.24, 1.04-1.47, <0.001). For *H19*, several studies investigated the association between SNPs and cancer risk, among which there were four studies about rs2107425 (Table 1). We then performed a meta-analysis to evaluate the association between rs2107425 and cancer risk (Table 2). Overall, we observed that rs2107425 could decrease the risk of cancer with the borderline effect in the dominat model (0.89, 0.80-0.99, 0.166), and no significant effect was found in other models (recessive model: 1.04, 0.94-1.16, 0.651; additive model: 0.92, 0.84-1.02, 0.173). For *POLR2E*, only one SNP rs3787016 had been investigated the association with cancer risk in the case-control studies. Meta-analysis result suggested the individuals with rs3787016 TT genotype had a 1.52-fold significantly increased cancer risk in the recessive model (1.52, 1.17-1.97, 0.201).

**Table 1:** Characteristics of the included studies

Gene	Authors	Year	Country	Ethnicity	Source of control	SNP	Cancer type	Genotyping method	Case	Control
***HOTAIR***	Yan et al.	2015	China	Asian	PB	rs1899663 G>T	Breast cancer	PCR–RFLP	502	504
						rs4759314 A>G			502	504
						rs920778 C>T			502	504
	Du et al.	2015	China	Asian	HB	rs4759314 A>G	Gastric cancer	TaqMan	1275	1644
	Pan et al.	2015	China	Asian	HB	rs1899663 G>T	Gastric cancer	PCR–RFLP	500	1000
						rs4759314 A>G			500	1000
						rs920778 C>T			800	1600
	Guo et al.	2015	China	Asian	HB	rs4759314 A>G	Gastric cancer	PCR–RFLP	515	654
	Bayram et al.	2015	Turky	Caucasian	HB	rs920778 C>T	Gastric cancer	TaqMan	104	209
	Bayram et al.	2015	Turky	Caucasian	HB	rs920778 C>T	Breast cancer	TaqMan	123	122
	Xue et al.	2014	China	Asian	HB	rs4759314 A>G	Colorectal cancer	TaqMan	1733	1855
	Zhang et al.	2014	China	Asian	HB	rs1899663 G>T	Esophageal cancer	PCR–RFLP	1000	1000
						rs4759314 A>G			1000	1000
						rs920778 C>T			2098	2150
***PRNCR1***	Li et al.	2015	China	Asian	HB	rs1016343 C>T	Gastric cancer	PCR–RFLP	219	394
						rs13252298 A>G			219	394
						rs7007694 T>C			219	394
						rs16901946 A>G			219	394
						rs1456315 A>G			219	394
	Hui et al.	2014	China	Asian	HB	rs1016343 C>T	Prostate cancer	PCR-HRM	284	284
						rs13252298 A>G			277	267
	Li et al.	2013	China	Asian	HB	rs1016343 C>T	Colorectal cancer	PCR–RFLP	313	595
						rs13252298 A>G		313	595	
						rs7007694 T>C			313	595
						rs16901946 A>G			313	595
						rs1456315 A>G			313	595
	Chung et al.	2011	Japan	Asian	HB	rs1016343 C>T	Prostate cancer	Multiplex PCR-based Invader assay	1502	1552
						rs13252298 A>G		1501	1550	
						rs7007694 T>C			1497	1554
						rs16901946 A>G			1504	1554
						rs1456315 A>G			1504	1553
	Zheng et al.	2010	China	Asian	HB	rs1016343 C>T	Prostate cancer	MassARRAY iPLEX system	284	147
	Salinas et al.	2008	USA	Caucasian	PB	rs1016343 C>T	Prostate cancer	SNPlex	1253	1233
				African Americans	PB	rs1016343 C>T	Prostate cancer	SNPlex	143	79
***H19***	Butt et al.	2012	Sweden	Caucasian	PB	rs2107425 C>T	Breast cancer	MALDI-TOF MS	679	1355
	Song et al.	2009	UK	Caucasian	PB	rs2107425 C>T	Ovarian cancer	TaqMan	5366	8538
	Verhaegh et al.	2008	Netherlands	Caucasian	PB	rs2107425 C>T	Bladder cancer	PCR-RFLP	177	204
	Bhatti et al.	2008	USA	Mixed	PB	rs2107425 C>T	Breast cancer	Unknown	824	1073
***POLR2E***	Kang et al.	2015	China	Asian	HB	rs3787016 C>T	Esophageal cancer	MALDI-TOF MS	369	370
	Cao et al.	2014	China	Asian	PB	rs3787016 C>T	Prostate cancer	TaqMan	1015	1032
	Nicolic et al.	2013	Serbia	Caucasian	HB	rs3787016 C>T	Prostate cancer	TaqMan	261	293
	Jin et al.	2011	USA	Caucasian	PB	rs3787016 C>T	Prostate cancer	Illumina chip, MassARRAY	4196	5007

PB: population based; HB: hospital based; PCR-RFLP: polymerase chain reaction restriction fragment length polymorphism; MALDI-TOF MS: matrix-assisted laser desorption/ionization time-of-flight mass spectrometry

**Table 2:** Pooled analyses of lncRNA polymorphims on cancer risk

Gene	SNPs	Allele (major/minor)	n^a^	Cases	Controls	Dom model		Rec model		Add model	
						OR (95% CI)	*P*^b^	OR (95% CI)	*P*^b^	OR (95% CI)	*P*^b^
***HOTAIR***	rs1899663	G>T	3	2002	2504	0.94 (0.82-1.07)	0.714	0.75 (0.49-1.12)	0.891	0.93 (0.83-1.04)	0.774
	rs4759314	A>G	6	5525	6657	1.05 (0.86-1.28)	0.024	0.75 (0.39-1.41)	0.880	1.04 (0.86-1.25)	0.027
	rs920778	C>T	5	3627	4585	1.20 (0.92-1.57)	0.005	1.61 (1.08-2.41)	<0.001	1.24 (1.03-1.49)	0.001
***PRNCR1***	rs1016343	C>T	7	3998	4284	1.27 (1.04-1.53)	0.002	1.33 (0.92-1.92)	<0.001	1.24 (1.04-1.47)	<0.001
	rs13252298	A>G	4	2310	2806	0.84 (0.55-1.28)	<0.001	0.89 (0.63-1.27)	0.059	0.89 (0.65-1.22)	<0.001
	rs7007694	T>C	3	2029	2543	0.93 (0.71-1.22)	0.027	1.10 (0.63-1.89)	0.053	0.96 (0.74-1.25)	0.008
	rs16901946	A>G	3	2036	2543	1.15 (1.02-1.29)	0.573	0.92 (0.37-2.26)	0.002	1.09 (0.91-1.30)	0.080
	rs1456315	A>G	3	2036	2542	0.70 (0.46-1.05)	<0.001	0.64 (0.31-1.31)	0.002	0.74 (0.53-1.04)	<0.001
***H19***	rs2107425	C>T	4	7046	11170	0.89 (0.80-0.99)	0.166	1.04 (0.94-1.16)	0.651	0.92 (0.84-1.02)	0.173
***POLR2E***	rs3787016	C>T	4	5841	6702	0.87 (0.61-1.26)	0.005	1.52 (1.17-1.97)	0.201	1.07 (0.94-1.22)	0.017

^a^ Number of comparisons.

^b^ *P*-value of Q-test for heterogeneity test.

Further, we evaluated the effects of lncRNA SNPs according to ethnicity and cancer type. We mainly focused on investigating the effects of three lncRNA polymorphisms (*HOTAIR* rs920778, *PRNCR1* rs1016343, and *POLR2E* rs3787016) in subgroups. As shown in Table 3, *HOTAIR* rs920778 contributed to the increased risk of esophageal cancer in all models (dominant model: 1.48, 1.31-1.67; recessive model: 2.51, 1.91-3.29; additive model: 1.48, 1.34-1.64). We also observed the similar effect in Asians (dominant model: 1.46, 1.33-1.61, 0.953; recessive model: 2.13, 1.42-3.20, 0.004; additive model: 1.46, 1.35-1.57, 0.803). For *PRNCR1* rs1016343, in stratified analyses by cancer type, increased risks were observed for prostate cancer in all models (dominant model: 1.39, 1.12-1.71, 0.012; recessive model: 1.83, 1.53-2.18, 0.855; additive model: 1.42, 1.29-1.56, 0.257). The similar associations were found in Caucasian populations (dominant model: 1.39, 1.18-1.63; recessive model: 1.72, 1.21-2.44; additive model: 1.36, 1.19-1.55). For *POLR2E*, we found that rs3787016 increased the risk of esophageal cancer (recessive model: 1.76, 1.25-2.49). In addition, elevated risk also was observed in Asian populations (recessive model: 1.64, 1.35-1.99, 0.624).

**Table 3:** Stratified analyses of the *HOTAIR* rs920778, *PRNCR1* rs1016343, *POLR2E* rs3787016 on cancer risk

Variables	n^a^	Cases	Controls	Dom model		Rec model		Add model	
				OR (95% CI)	*P*^b^	OR (95% CI)	*P*^b^	OR (95% CI)	*P*^b^
***HOTAIR* rs920778**	5	3627	4585	1.20 (0.92-1.57)	0.005	1.61 (1.08-2.41)	<0.001	1.24 (1.03-1.49)	0.001
**Cancer type**									
Breast cancer	2	625	626	0.79 (0.22-2.78)	0.012	1.26 (0.85-1.88)	0.156	1.03 (0.57-1.86)	0.005
Gastric cancer	2	904	1809	1.28 (0.89-1.85)	0.181	1.65 (0.59-4.61)	0.001	1.21 (0.82-1.80)	0.029
Esophageal cancer	1	2098	2150	1.48 (1.31-1.67)	-	2.51 (1.91-3.29)	-	1.48 (1.34-1.64)	-
**Ethnicity**									
Asian	3	3400	4254	1.46 (1.33-1.61)	0.953	2.13 (1.42-3.20)	0.004	1.46 (1.35-1.57)	0.803
Caucasian	2	227	331	0.63 (0.29-1.40)	0.080	0.96 (0.66-1.38)	0.976	0.86 (0.67-1.10)	0.321
***PRNCR1* rs1016343**	7	3998	4284	1.27 (1.04-1.53)	0.002	1.33 (0.92-1.92)	<0.001	1.24 (1.04-1.47)	<0.001
**Cancer Type**									
Colorectal cancer	1	313	595	1.03 (0.78-1.37)	-	0.80 (0.54-1.19)	-	0.96 (0.79-1.17)	-
Gastric cancer	1	219	394	1.00 (0.71-1.41)	-	0.69 (0.44-1.09)	-	0.90 (0.71-1.14)	-
Prostate cancer	5	3466	3295	1.39 (1.12-1.71)	0.012	1.83 (1.53-2.18)	0.855	1.42 (1.29-1.56)	0.257
**Ethnicity**									
African American	1	143	79	0.78 (0.45-1.36)	-	-	-	-	-
Asian	5	2602	2972	1.30 (1.00-1.69)	0.002	1.26 (0.80-1.97)	<0.001	1.21 (0.96-1.53)	<0.001
Caucasian	1	1253	1233	1.39 (1.18-1.63)	-	1.72 (1.21-2.44)	-	1.36 (1.19-1.55)	-
***POLR2E* rs3787016**	4	5841	6702	0.87 (0.61-1.26)	0.005	1.52 (1.17-1.97)	0.201	1.07 (0.94-1.22)	0.017
**Cancer Type**									
Esophageal cancer	1	369	370	0.73 (0.53-0.99)	-	1.76 (1.25-2.49)	-	0.97 (0.79-1.19)	-
Prostate cancer	3	5472	6332	0.95 (0.59-1.53)	0.012	1.32 (0.81-2.16)	0.112	1.10 (0.95-1.27)	0.025
**Ethnicity**									
Asian	2	1384	1402	0.95 (0.59-1.52)	0.008	1.64 (1.35-1.99)	0.624	1.08 (0.90-1.31)	0.116
Caucasian	2	4457	5300	0.73 (0.52-1.02)	-	0.94 (0.51-1.72)	-	1.01 (0.70-1.44)	0.007

^a^ Number of comparisons.

^b^ *P*-value of Q-test for heterogeneity test.

### Meta-regression

We observed the significant heterogeneity between studies in the meta-analysis. Then, we performed the meta-regression analysis to assess the source of heterogeneity in the additive model (Table 4). For *HOTAIR* rs920778, we observed that ethnicity and genotyping method were the source of heterogeneity (all *P*< 0.001), and could explain 100% of the Tau-squared. Similarly, we also found that the source of control (*P* = 0.002) and genotyping method (*P* = 0.047) could explain the 100% of the Tau-squared for the *POLR2E* rs3787016. Additionally, for the *PRNCR1* rs1016343, cancer type (*P* <0.001) was observed to contribute to substantial heterogeneity (95% of the Tau-squared).

**Table 4:** Meta-regression analysis of the *HOTAIR* rs920778, *PRNCR1* rs1016343, *POLR2E* rs3787016 on cancer risk (additive model)

SNPs	Variables	Coefficient	Standard error	*P*	95% CI
*HOTAIR* rs920778	Ethnicity	0.529	0.131	<0.001	0.27-0.79
	Source of control	0.168	0.349	0.631	−0.52-0.85
	Cancer type	0.076	0.181	0.672	−0.28-0.43
	Genotyping method	0.529	0.131	<0.001	0.27-0.79
*PRNCR1* rs1016343	Ethnicity	0.117	0.272	0.667	−0.42-0.65
	Source of control	0.117	0.272	0.667	−0.42-0.65
	Cancer type	0.263	0.063	<0.001	0.14-0.39
	Genotyping method	0.097	0.055	0.078	−0.01-0.20
*POLR2E* rs3787016	Ethnicity	−0.053	0.202	0.795	−0.45-0.34
	Source of control	0.265	0.087	0.002	0.09-0.44
	Cancer type	0.107	0.219	0.627	−0.32-0.54
	Genotyping method	0.092	0.046	0.047	0.00-0.18

### Publication bias

**Figure 2:**
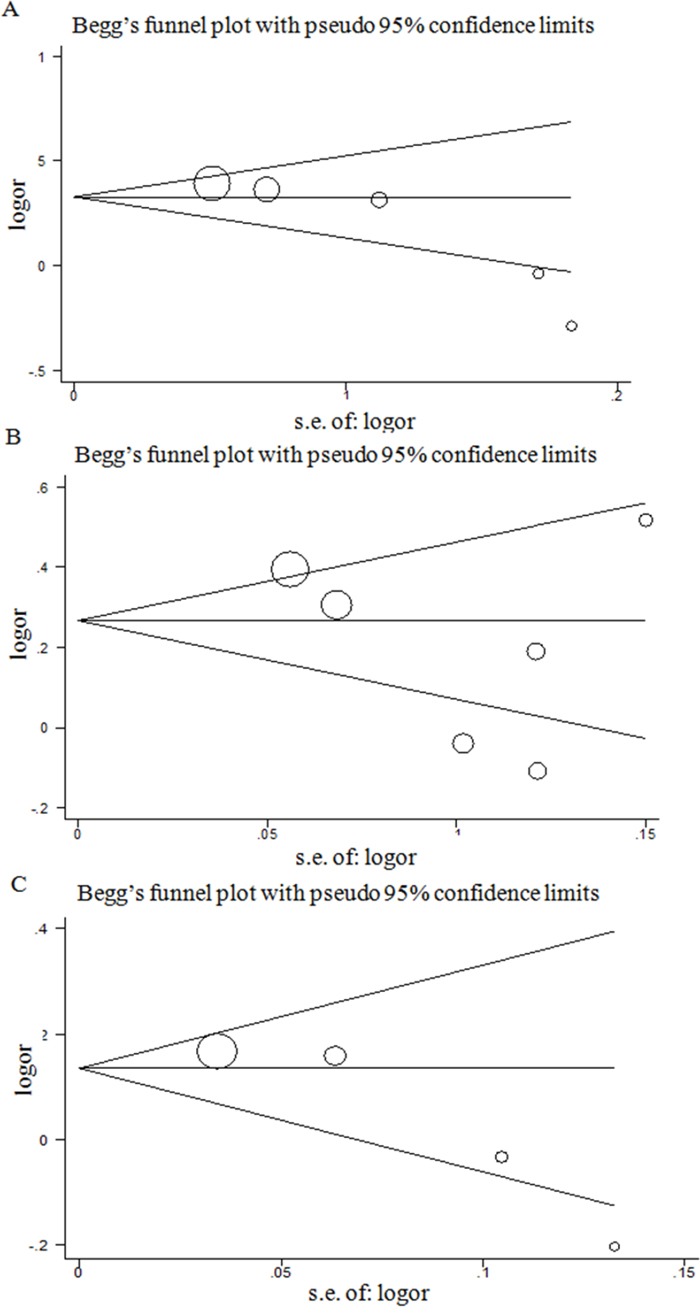
Begg's funnel plot for publication bias test (additive model) Each point represents a separate study for the indicated association. Log[or], natural logarithm of OR. Horizontal line, mean effect size. **A**. *HOTAIR* rs920778. **B**. *PRNCR1* rs1016343. **C**. *POLR2E* rs3787016.

Egger's test and Begg's test were used to evaluate the publication bias of the *HOTAIR* rs920778, *PRNCR1* rs1016343, *POLR2E* rs3787016 in the meta-analysis. Egger's test results demonstrated that there was a significant publication bias in the additive model for the *HOTAIR* rs920778 (*t* = -3.91, *P* = 0.030), and no significant publication bias was found for the other two SNPs (*PRNCR1* rs1016343: *t* = -1.16, *P* = 0.311; *POLR2E* rs3787016: *t* = -3.20, *P* = 0.085) (Figure 2). Begg's funnle plot also showed the asymmetry for the *HOTAIR* rs920778, suggesting the existence of publication bias (Figure 2A). Furthermore, we used a trim-and-fill method to adjust the bias, which was developed by Duval and Tweedie [35]. The adjusted result from the random model was ORs of 1.26 (1.06–1.46) for *HOTAIR* rs920778 in the additive model, which was similar with our results (OR = 1.24, 95%CI = 1.03-1.49).

## DISCUSSION

LncRNAs participate in the diverse biological process, and abnormal expression of lncRNAs is associated with human cancers [36, 37]. Cumulative studies have suggested that lncRNAs polymorhisms have been widely studied, and are associated with cancer risk. However, results are not consistent. Thus, it is warranted to identify the association between lncRNAs polymorphisms and cancer risk. Through searching the PubMed, we screen the all published articles, and finally, we include the lncRNA *HOTAIR*, *PRNCR1*, *H19*, and *POLR2E* polymorphisms in this meta-analysis. The results indicate that these four lncRNAs polymorphisms (*HOTAIR* rs920778, *PRNCR1* rs1016343 and rs16901946, *POLR2E* rs3787016) can contribute to the increased risk of cancer.

HOTAIR can bind to the polycomb-repressive complex 2 [5] and lysine specific demethylase 1 complex [38] to play the roles in cell biological process. At present, *HOTAIR* polymorphisms are the most commonly studied than other lncRNAs in human diseases, particularly in cancer. Interestingly, in recent two years, there have nine studies to investigate the association between *HOTAIR* polymorphisms and cancer risk. The polymorphisms in *HOTAIR* will be a hot concern in predicting cancer risk. For example, Yan et al. and his colleagues proposed that *HOTAIR* rs920778 improved the risk of breast cancer in Chinese populations [14]. In the esophageal cancer, authors also observed the similar result [20]. However, in a Turkish population, no significant association was found in gastric cancer [17]. Our meta-analysis results indicated that rs920778 can increase the cancer susceptibility. In the subgroup analysis, we also observed the significant improved risk in Asians and in esophageal cancer. Further, we performed the combination analysis of HaploReg and RegulomeDB to annotate the function of *HOTAIR* rs920778. As shown in Supplementary Table 2, we found that rs920778 were related to DNase I hypersensitivity and DNA-binding motifs. For Asians, the C allele frequency was 0.76, which was higher than in Caucasians (European) (0.69). Difference in genetic background may reflect the results. Herein, only one included study was involved in esophageal cancer [20], thus, we should interpret the findings with caution. Other common SNPs in *HOTAIR* (rs1899663 and rs4759314) also had been included in this meta-analysis, and no significant association is present.

Besides, we also investigated the effect of lncRNA PRNCR1 on cancer risk. PRNCR1 also known as PCAT8, was highly expressed in prostate cancer, and can mediate the prostate cancer cells gene activation programs and proliferation by binding to the androgen receptor [39]. Chung et al. used the resequencing and fine mapping of 8q24 region (Chr8: 128.14-128.28 Mb) and confirmed the *PRNCR1* polymorphisms associated with prostate cancer risk [24]. Meanwhile, genetic variations in *PRNCR1* also had been investigated in gastric cancer [22] and colorectal cancer [23]. In order to comprehensively evaluate the precise effect of *PRNCR1* rs1016343 on cancer risk, we performed a meta-analysis to summarize all published studies. Results indicated that rs1016343 indeed can predict the cancer susceptibility, particularly in prostate cancer and Caucasians. It was worth to note that the published studies were mainly involved in prostate cancer and Asians, thus, other cancer types and ethnicity should be further investigated to validate these findings. Functional annotation suggested rs1016343 exhibiting DNase I hypersensitivity, Protein binding and DNA-binding motifs (Supplementary Table 2), which may be related with PRNCR1 expression. Herein, we also evaluated the association of *PRNCR1* rs13252298, rs7007694, rs16901946 and rs1456315 and cancer risk in this meta-analysis. The pooled results suggested that *PRNCR1* rs16901946 was associated with increased risk of cancer, which was consistent with Chung et al. findings in prostate cancer [24]. We noticed that there were three studies regarding with rs16901946. However, rs16901946 was not associated with gastric cancer and colorectal cancer risk [22–24]. In the future, more studies should be conducted to evaluate the effect of rs16901946 on cancer risk, such as prostate cancer, gastric cancer, colorectal cancer, etc.

Comparing with the above two lncRNA genes, *POLR2E* polymorphism was relatively less studied in cancer. So far, there are only four studies to investigate the association between *POLR2E* rs3787016 and cancer risk. Jin et al firstly reported the rs3787016 in *POLR2E* gene that was associated with prostate cancer susceptibility based on prostate cancer genome wide association study [7]. Subsequently, authors investigated the role of rs3787016 in esophageal cancer [33] and prostate cancer [8, 34]. Meta-analysis results revealed that rs3787016 can predict the cancer risk. In the stratification analysis of cancer type, we observed that rs3787016 was associated with the risk of esophageal cancer, but not prostate cancer. We found that among the included four studies, three studies investigated the association between rs3787016 and prostate cancer risk [7, 8, 34], however, only Jin et al. identified the significant SNP rs3787016 with prostate cancer risk [7]. In addition, we observed that the population of included studies had both Asian and Caucasian populations. In the stratification analysis of ethnicity, there was significant association between rs3787016 and cancer risk in Asian population. Differences in genetic background may be a possible reflection of rs3787016 on cancer risk. Therefore, larger studies were warranted to be performed in prostate cancer and different ethnicity.

We also summarized the results of the lncRNA *H19* polymorphisms with cancer risk. As early as 1990, Brannan et al. had identified the function of H19 gene as an RNA [40]. Aberrant expression of H19 had participated in multiple cancers [41, 42]. There were many studies to investigate the SNPs within *H19* associated with cancer susceptibility [27–32]. Bhatti et al. found that *H19* rs2107425 was not associated with overall breast cancer risk, however, individuals with the *H19* rs2107425 variant alleles had the decreased risk of breast cancer in occupational radiation low-dose group and with the increased breast cancer risk in high-dose group [30]. In another population-based prospective cohort, we observed that rs2107425 variant alleles were associated with decreased risk of breast cancer [31]. Additionally, the effect of *H19* rs2107425 also was investigated on the risk of bladder cancer and ovarian cancer. In bladder cancer, authors found the borderline effect for rs2107425 polymorphism [29], while in ovarian cancer, we did not observe the significant effect for rs2107425 polymorphism [32]. Therefore, we performed a meta-analysis to summarize the results of rs2107425 and cancer risk. The pooled results showed that *H19* rs2107425 had the borderline effect on cancer risk. Further studies should validate our findings.

Due to the identification of large number of lncRNAs, aberrant expression of lncRNAs have been widely studied, and several meta-analysis have been done to evaluate the expression of lncRNAs and the prognosis of cancer [36, 43]. At present, there have no study to systematically investigate the association between lncRNAs polymorphisms and cancer risk. Herein, we focused on exploring the common lncRNAs polymorphisms associated with cancer risk in a meta-analysis. Interestingly, we indeed found the lncRNAs polymorphisms were related with cancer risk. These findings can provide the useful information to support the further study in the function of lncRNAs polymorphisms. However, we also noticed that among the included studies were shown the significant heterogeneity. Meta-regression revealed that the source of heterogeneity mainly from the ethnicity, genotyping method, cancer type, and source of control, suggesting these four factors playing the crucial roles.

Although we observed the significant association between lncRNAs polymorphisms and cancer risk in this meta-analyais, however, several limitations should be warranted. Due to lacking the detailed data of included studies, our evaluation mainly focused on unadjusted results of lncRNAs polymorphisms and cancer risk. Additionally, our meta-analysis included several small sample size studies, which may influence the results stability. It was worth to note that the majority of studies matching with age, sex and residential environment can control the selection bias.

In conclusion, our meta-analysis showed that lncRNA *HOTAIR* rs920778, *PRNCR1* rs1016343 and rs16901946, *POLR2E* rs3787016 were associated with cancer susceptibility. However, due to several confounding factors, we should explain the results with caution. Further larger and multi-ethnicity studies should confirm our findings. Moreover, genetic factors should combine with environmental factors to predict the risk of cancer, which will lead to comprehensively understand the association between lncRNAs polymorphisms and cancer risk.

## MATERIALS AND METHODS

### Study selection

We performed a comprehensive article search in PubMed up to February 2016 using the key words ‘lncRNA’ or ‘long non-coding RNA’ and ‘polymorphism’ to summarize the articles of relevant lncRNA polymorphisms and cancer risk. Besides, we also used the manual search of the references of relavant articles. The included studies should meet some criteria: firstly, studies should be written in English language; secondly, studies should be a human case-control design; thirdly, studies had to report the genotypes frequency. We also had some excluded criteria: firstly, only one or two studies investigated one particular lncRNA gene, which was not suitable for meta-analysis; secondly, two studies used the same data, and we chose the study with largest sample size.

### Data extraction

Two authors (Chu H and Chen Y) independently extracted the available data from the selected studies, and data should be crosschecked. We mainly focused on collecting the following data: first author's name, year of publication, country, ethnicity, source of control (hospital-based or population-based), genotyping method, genotype frequency of SNPs, case and control numbers, matching factors, Hardy-Weingberg equilibrium (HWE) and cancer type. We categorized ethnicity as Asian, Caucasian, or African decent population. If one study was composed of multiple ethnic populations and we can not distinguish the different ethnicity among the multiple ethnic populations, we named it mixed population [30]. For study including Caucasian and African decent population (Salinas et al., 2008), we separately extracted the data for each ethnic group [6]. There were three studies without data for all three genotypes, therefore, we calculated the odds ratios (ORs) for dominant model [6, 30] and additive model [7] in statistical analysis.

### Statistical analysis

In this meta-analysis, we mainly used the ORs and 95% confidence intervals (CIs) to investigate the association between lncRNA polymorphism and cancer risk. For lncRNA polymorphisms, we calculated the risks of the dominant model, recessive model and additive model (linearly according to 0, 1, 2 minor allele). For example, A is the wild allele, and B is the variant allele. In the dominant model, we estimated the risk of the variant genotype AB and BB, compared with wild genotype AA (AB/BB versus AA), and evaluated the risk of BB versus AB/AA, assuming recessive effects of the variant B allele. It should be mentioned that there were two studies without data for all three genotypes [6, 7], therefore, we calculated the results in recessive model and additive model, respectively. In addition, we also performed the stratified analyses of ethnicity and cancer type.

The meta-analysis of included studies was conducted with a random-effect model (the DerSimonian and Laird method), which mainly considered the variability within or between study [44]. The Cochran's Q-test and I^2^ were used for assessment of studies heterogeneity, and heterogeneity was supposed to be significant when *P* value was less than 0.10 [45]. In this study, we further examined the ethnicity, source of control and genotyping methods in meta-regression model to explore the source of heterogeneity. The Tau-squared was used to estimate of between study variance and the less *P*_heterogeneity_ value was, the more tau-squared was [46]. We always used the Begg's and Egger's test to assess the publication bias of included studies. Publication bias was shown, when *P* value was less than 0.05. HaploReg (v4.1) and RegulomeDB (v1.1) were used to annotate the function of SNPs.

All statistical analyses were conducted with the STATA 12.0 (StataCorp, College Station, TX). A *P* value< 0.05 was considered significantly.

## SUPPLEMENTARY TABLES




