# Supplementary material for: The HOTAIR, PRNCR1 and POLR2E polymorphisms are associated with cancer risk: a meta-analysis
Source: Oncotarget. 2017 Jan 31;8(26):43271–83. doi: 10.18632/oncotarget.14920 (PMC5522144; doi:10.18632/oncotarget.14920)
Supplement: Supplementary file 1 [file oncotarget-08-43271-s001.pdf]

The HOTAIR, PRNCR1 and POLR2E polymorphisms are associated with cancer risk: a meta-analysis

SUPPLEMENTARY MATERIALS

Supplementary Table 1: Characteristics of the included studies (all SNPs)

See Supplementary File 1

Supplementary Table 2: Functional annotation of SNPs at *HOTAIR, PRNCR1,* and *POLR2E* based on HaploReg and Regulome DB

See Supplementary File 1
