# Supplementary material for: The HOTAIR, PRNCR1 and POLR2E polymorphisms are associated with cancer risk: a meta-analysis
Source: Oncotarget. 2017 Jan 31;8(26):43271–83. doi: 10.18632/oncotarget.14920 (PMC5522144; doi:10.18632/oncotarget.14920)
Supplement: Supplementary file 2 [file oncotarget-08-43271-s002.docx]

**Supplementary Table 1: Characteristics of the included studies (all SNPs)**

| **Gene** | **Authors** | **Year** | **Country** | **Ethnicity** | **Source of control** | **SNP** | **Cancer type** | **Genotyping method** | **Case** | **Control** |
| --- | --- | --- | --- | --- | --- | --- | --- | --- | --- | --- |
| ***HOTAIR*** | Yan et al. | 2015 | China | Asian | PB | rs1899663 G>T | Breast cancer | PCR–RFLP | 502 | 504 |
|  |  |  |  |  |  | rs4759314 A>G |  |  | 502 | 504 |
|  |  |  |  |  |  | rs920778 C>T |  |  | 502 | 504 |
|  | Du et al. | 2015 | China | Asian | HB | rs4759314 A>G | Gastric cancer | TaqMan | 1275 | 1644 |
|  |  |  |  |  |  | rs7958904 G>C |  |  | 739 | 1057 |
|  |  |  |  |  |  | rs874945 G>A |  |  | 751 | 1057 |
|  | Pan et al. | 2015 | China | Asian | HB | rs1899663 G>T | Gastric cancer | PCR–RFLP | 500 | 1000 |
|  |  |  |  |  |  | rs4759314 A>G |  |  | 500 | 1000 |
|  |  |  |  |  |  | rs920778 C>T |  |  | 800 | 1600 |
|  | Guo et al. | 2015 | China | Asian | HB | rs12826786 C>T | Gastric cancer | PCR–RFLP | 515 | 654 |
|  |  |  |  |  |  | rs4759314 A>G |  |  | 515 | 654 |
|  |  |  |  |  |  | rs10783618 C>T |  |  | 515 | 654 |
|  | Bayram et al. | 2015 | Turky | Caucasian | HB | rs12826786 C>T | Breast cancer | TaqMan | 123 | 122 |
|  | Bayram et al. | 2015 | Turky | Caucasian | HB | rs920778 C>T | Gastric cancer | TaqMan | 104 | 209 |
|  | Bayram et al. | 2015 | Turky | Caucasian | HB | rs920778 C>T | Breast cancer | TaqMan | 123 | 122 |
|  | Xue et al. | 2014 | China | Asian | HB | rs4759314 A>G | Colorectal cancer | TaqMan | 1733 | 1855 |
|  |  |  |  |  |  | rs7958904 G>C |  |  | 1731 | 1852 |
|  |  |  |  |  |  | rs874945 G>A |  |  | 1147 | 1202 |
|  | Zhang et al. | 2014 | China | Asian | HB | rs1899663 G>T | Esophageal cancer | PCR–RFLP | 1000 | 1000 |
|  |  |  |  |  |  | rs4759314 A>G |  |  | 1000 | 1000 |
|  |  |  |  |  |  | rs920778 C>T |  |  | 2098 | 2150 |

**Supplementary Table 1. Continued**

| **Gene** | **authors** | **Year** | **Country** | **Ethnicity** | **Source of control** | **SNP** | **Cancer type** | **Genotyping method** | **Case** | **Control** |
| --- | --- | --- | --- | --- | --- | --- | --- | --- | --- | --- |
| ***PRNCR1*** | Li et al. | 2015 | China | Asian | HB | rs1016343 C>T | Gastric cancer | PCR–RFLP | 219 | 394 |
|  |  |  |  |  |  | rs13252298 A>G |  |  | 219 | 394 |
|  |  |  |  |  |  | rs7007694 T>C |  |  | 219 | 394 |
|  |  |  |  |  |  | rs16901946 A>G |  |  | 219 | 394 |
|  |  |  |  |  |  | rs1456315 A>G |  |  | 219 | 394 |
|  | Hui et al. | 2014 | China | Asian | HB | rs1016343 C>T | Prostate cancer | PCR-HRM | 284 | 284 |
|  |  |  |  |  |  | rs13252298 A>G |  |  | 277 | 267 |
|  | Li et al. | 2013 | China | Asian | HB | rs1016343 C>T | Colorectal cancer | PCR–RFLP | 313 | 595 |
|  |  |  |  |  |  | rs13252298 A>G |  |  | 313 | 595 |
|  |  |  |  |  |  | rs7007694 T>C |  |  | 313 | 595 |
|  |  |  |  |  |  | rs16901946 A>G |  |  | 313 | 595 |
|  |  |  |  |  |  | rs1456315 A>G |  |  | 313 | 595 |
|  | Chung et al. | 2011 | Japan | Asian | HB | rs1016343 C>T | Prostate cancer | Multiplex PCR-based Invader assay | 1502 | 1552 |
|  |  |  |  |  |  | rs13252298 A>G |  |  | 1501 | 1550 |
|  |  |  |  |  |  | rs7007694 T>C |  |  | 1497 | 1554 |
|  |  |  |  |  |  | rs16901946 A>G |  |  | 1504 | 1554 |
|  |  |  |  |  |  | rs1456315 A>G |  |  | 1504 | 1553 |
|  | Zheng et al. | 2010 | China | Asian | HB | rs1016343 C>T | Prostate cancer | MassARRAY iPLEX system | 284 | 147 |
|  | Salinas et al. | 2008 | USA | Caucasian | PB | rs1016343 C>T | Prostate cancer | SNPlex | 1253 | 1233 |
|  |  |  |  | African Americans | PB | rs1016343 C>T | Prostate cancer | SNPlex | 143 | 79 |

**Supplementary Table 1. Continued**

| **Gene** | **authors** | **Year** | **Country** | **Ethnicity** | **Source of control** | **SNP** | **Cancer type** | **Genotyping method** | **Case** | **Control** |
| --- | --- | --- | --- | --- | --- | --- | --- | --- | --- | --- |
| ***H19*** | Yang et al. | 2015 | China | Asian | HB | rs217727 C>T | Gastric cancer | TaqMan | 500 | 500 |
|  |  |  |  |  |  | rs2839698 C>T |  |  | 500 | 500 |
|  |  |  |  |  |  | rs3741216 A>T |  |  | 500 | 500 |
|  |  |  |  |  |  | rs3741219 T>C |  |  | 500 | 500 |
|  | Butt et al. | 2012 | Sweden | Caucasian | PB | rs2107425 C>T | Breast cancer | MALDI-TOF MS | 679 | 1355 |
|  | Soares et al. | 2010 | Brazil | Mixed | HB | RsaI A>G | Melanoma | PCR-RFLP | 21 | 100 |
|  | Song et al. | 2009 | UK | Caucasian | PB | rs2107425 C>T | Ovarian cancer | TaqMan | 5366 | 8538 |
|  | Verhaegh et al. | 2008 | Netherlands | Caucasian | PB | rs217727 C>T | Bladder cancer | PCR-RFLP | 177 | 204 |
|  |  |  |  |  |  | rs2839698 C>T |  |  | 177 | 204 |
|  |  |  |  |  |  | rs2107425 C>T |  |  | 177 | 204 |
|  |  |  |  |  |  | rs2735469 C>T |  |  | 177 | 204 |
|  |  |  |  |  |  | rs17658052 G>A |  |  | 177 | 204 |
|  | Bhatti et al. | 2008 | USA | Mixed | PB | rs2107425 C>T | Breast cancer | Unknown | 824 | 1073 |
| ***POLR2E*** | Kang et al. | 2015 | China | Asian | HB | rs3787016 C>T | Esophageal cancer | MALDI-TOF MS | 369 | 370 |
|  | Cao et al. | 2014 | China | Asian | PB | rs3787016 C>T | Prostate cancer | TaqMan | 1015 | 1032 |
|  | Nicolic et al. | 2013 | Serbia | Caucasian | HB | rs3787016 C>T | Prostate cancer | TaqMan | 261 | 293 |
|  | Jin et al. | 2011 | USA | Caucasian | PB | rs3787016 C>T | Prostate cancer | Illumina chip, MassARRAY | 4196 | 5007 |

PB: population based; HB: hospital based; PCR-RFLP: polymerase chain reaction restriction fragment length polymorphism; MALDI-TOF MS: matrix-assisted laser desorption/ionization time-of-flight mass spectrometry.

**Supplementary Table 2: Functional annotation of SNPs at *HOTAIR*, *PRNCR1*, and *POLR2E* based on HaploReg and Regulome DB**

| **Chr** | **Pos (hg19)** | **SNP** | **Ref** | **Alt** | **Frequency** | | | | **Promoter**  **histone marks** | **Enhancer**  **histone marks** | **DNAse** | **Proteins**  **bound** | **Motifs changed** | **GENCODE**  **genes** | **dbSNP func annot** | **Regulome**  **DB score*** |
| --- | --- | --- | --- | --- | --- | --- | --- | --- | --- | --- | --- | --- | --- | --- | --- | --- |
|  |  |  |  |  | **AFR** | **AMR** | **ASN** | **EUR** |  |  |  |  |  |  |  |  |
| 12 | 53966448 | [rs920778](http://www.broadinstitute.org/mammals/haploreg/detail_v4.1.php?query=&id=rs920778) | C | T | 0.31 | 0.58 | 0.76 | 0.69 | 12 tissues | 11 tissues | 13 tissues |  | DMRT4,DMRT5,THAP1 | HOTAIR | intronic | 2b |
| 8 | 127081052 | [rs1016343](http://www.broadinstitute.org/mammals/haploreg/detail_v4.1.php?query=&id=rs1016343) | C | T | 0.18 | 0.15 | 0.30 | 0.21 |  |  | 18 tissues | CFOS,PRDM1,  STAT3 | 5 altered motifs | RP11-255B23.2  (PRNCR1) |  | 3a |
| 19 | 1090804 | [rs3787016](http://www.broadinstitute.org/mammals/haploreg/detail_v4.1.php?query=&id=rs3787016) | A | G | 0.87 | 0.81 | 0.53 | 0.75 |  | 5 tissues | 4 tissues |  | E2A,Ik-1,Zfp187 | POLR2E | 3'-UTR | 5 |

Regulome DB score: 3a, TF binding + any motif + DNase peak; 3b, TF binding + matched TF motif; 4, TF binding + DNase peak; 5, TF binding or DNase peak; 6, motif hit; 7, no data supporting.
